# The Impact of CT-Guided Cordotomy on Severe Cancer Pain

**DOI:** 10.3390/jcm15218270

**Published:** 2026-10-23

**Authors:** Gozde Dagistan, Fatih Kursad Bahcivan, Nurten Kayacan, Mert Akbas, Bilge Karsli

**Affiliations:** 1 Division of Algology, Department of Anesthesiology, Medical Faculty, Akdeniz University, 07070 Antalya, Turkey; nurtenkayacan@akdeniz.edu.tr (N.K.); bilgekarsli@akdeniz.edu.tr (B.K.); 2 Department of Anesthesiology, Medical Faculty, Akdeniz University, 07070 Antalya, Turkey; fatihbahcivan94@gmail.com; 3 Private Pain Management Clinic, 07070 Antalya, Turkey

**Keywords:** cancer pain, cordotomy, palliative care, opioid, VAS

## Abstract

**Background:** Severe cancer-related pain remains a major clinical challenge in patients with advanced malignancies and may persist despite multimodal analgesic treatment. **Objective:** This study evaluated short-term clinical outcomes and complications associated with computed tomography (CT)-guided percutaneous cordotomy for severe, unilateral cancer-related pain. **Design:** This retrospective single-center case series included 21 patients with treatment-refractory cancer pain who underwent CT-guided cordotomy between 2019 and 2024. Pain intensity, functional status, and opioid consumption were assessed at baseline and at the standardized 1-month follow-up using the Visual Analog Scale (VAS), Karnofsky Performance Status (KPS), and daily morphine milligram equivalents (MME), respectively. **Results:** At 1 month, the mean VAS score decreased from 9.95 to 0.90 (*p* < 0.001), mean daily MME decreased from 876.00 to 205.62 mg/day (*p* < 0.001), and mean KPS increased from 38.10 to 69.52 (*p* < 0.001). No permanent or major complications were documented during the available 1-month follow-up. **Conclusions**: In this retrospective single-center case series, CT-guided percutaneous cordotomy was associated with marked short-term reductions in pain intensity and opioid requirements and improvement in functional status at 1 month in patients with severe, unilateral cancer pain refractory to conventional therapy. Given the small sample size, absence of a control group, and limited follow-up duration, these findings should be considered preliminary and should not be interpreted as establishing definitive efficacy or safety.

## 1. Introduction

The management of severe cancer pain remains a major challenge in oncology, significantly impairing quality of life in patients with advanced malignancies. Cancer pain is complex and influenced by disease stage, tumor location, metastatic spread, and individual patient factors, making effective treatment difficult. Consequently, many patients experience pain that remains refractory despite evidence-based strategies, including the World Health Organization’s three-step analgesic ladder. This is particularly evident when opioid therapy fails to provide adequate analgesia or is limited by adverse effects. In such cases, specialized interventional techniques may be considered to improve pain control while reducing reliance on opioids.

Within the spectrum of interventional pain management strategies, neurosurgical techniques targeting specific neuroanatomical pathways may be considered for patients with treatment-refractory cancer pain. Among these interventions, percutaneous cordotomy performed under computed tomography (CT) guidance is an option for patients with unilateral, anatomically localized cancer pain that does not respond to conventional multimodal analgesic treatment and may reduce pain and systemic opioid requirements [1,2,3,4,5].

The present study evaluated short-term clinical outcomes and complications associated with CT-guided percutaneous cordotomy in patients with severe, treatment-refractory cancer pain. Outcomes included pain intensity measured using the Visual Analog Scale (VAS), functional status assessed using the Karnofsky Performance Status (KPS), and opioid consumption quantified as morphine milligram equivalents (MME).

## 2. Materials and Methods

### Study Design

This retrospective case series (Ethics approval: TBAEK-11/02.01.2025) analyzed the clinical data of 21 patients who underwent CT-guided percutaneous cordotomy for treatment-refractory cancer-related pain at the University Hospital’s Algology Clinic. The ethics committee approval covered the retrospective use and analysis of clinical data. All patients had provided written informed consent prior to the cordotomy procedure using the institutional consent form valid at the time of treatment, which included authorization for the scientific use of clinical data provided that patient identity remained confidential. All data included in the present study were de-identified, and no personally identifiable information was included in the study database or manuscript. The supplementary consent form submitted with this manuscript is the current institutional version, revised on 17 April 2026, and is provided only to illustrate the institution’s general consent framework regarding the use of de-identified clinical data for scientific research. It should not be interpreted as the specific contemporaneous consent form signed by the patients included in this retrospective case series.

This retrospective case series comprised all consecutive eligible patients with treatment-refractory cancer pain who underwent CT-guided percutaneous cordotomy between 2019 and 2024. The study included patients with unilateral cancer-related pain localized below the C5 dermatome, a baseline pain score ≥5 on the Visual Analog Scale (VAS), and inadequate pain control despite medical treatment and interventional pain procedures other than cordotomy. Patients with incomplete medical records were excluded. All patients underwent preoperative clinical evaluation for pulmonary reserve, coagulation status, and other contraindications as part of routine practice before being offered cordotomy. Patients with insufficient pulmonary reserve or thrombocytopenia were not referred for the procedure. Twenty-three patients were initially identified; two were excluded because of incomplete medical records, yielding a final analytical cohort of 21 patients. The cordotomy procedures were performed using the standardized technique described by Yücel Kanpolat.

Patients were monitored for 5–6 h after the procedure and discharged in the absence of immediate complications. Clinical outcomes were evaluated using the KPS for functional status and the VAS for pain intensity. VAS and KPS scores and opioid requirements were recorded at baseline and at the 1-month post-procedural follow-up. VAS was used to assess current pain intensity at the time of evaluation, using the same assessment condition at baseline and follow-up. Opioid consumption was quantified as morphine milligram equivalents (MME) to assess changes in analgesic requirements following the intervention. MME values were calculated using the equianalgesic conversion factors published in the NCCN Adult Cancer Pain Guidelines, with reference to the systematic review by Mercadante and Caraceni on opioid switching [6,7]. Treatment-refractory pain was defined as persistent severe cancer-related pain despite ongoing pharmacological treatment, including opioid therapy, and/or previous interventional pain management procedures, when applicable. No predefined minimum opioid dose, mandatory combination of analgesic classes, fixed treatment duration, or requirement for a specific prior interventional procedure was used as an eligibility criterion. The decision to proceed with cordotomy was based on inadequate pain control despite available treatments and clinical assessment by the treating pain physicians. Post-procedural opioid dose reduction was not based on a standardized tapering protocol; instead, opioid tapering was individualized by the treating clinicians according to each patient’s pain relief, functional status, opioid requirements, and overall clinical condition.

Procedure-related adverse events were retrospectively identified from the medical records, including procedure notes, inpatient/outpatient follow-up records, and the standardized 1-month follow-up assessment. Adverse events documented from the time of the procedure through the 1-month follow-up were recorded.

## 3. Results

A total of 21 patients were included in the study. The mean age of the patients was 57.81 ± 10.84 years, with a median age of 60 years (IQR: 50–65) and an age range of 37–71 years. Before cordotomy, the median KPS was 40 (IQR: 30–40; range: 20–60), with a mean of 38.10 ± 11.23. After cordotomy, the median KPS increased to 70 (IQR: 60–80; range: 50–90), with a mean of 69.52 ± 11.61. The median pre-cordotomy VAS score was 10 (IQR: 10–10; range: 9–10), with a mean of 9.95 ± 0.22, whereas after the procedure, the median VAS score decreased to 1 (IQR: 0–1; range: 0–2), with a mean of 0.90 ± 0.77. Similarly, daily opioid consumption decreased from a pre-cordotomy median of 858 MME/day (IQR: 754–960; range: 618–1320) and a mean of 876.00 ± 153.59 MME/day to a post-cordotomy median of 198 MME/day (IQR: 160–240; range: 120–360) and a mean of 205.62 ± 60.46 MME/day.

Of the 21 patients included in the study, 14 (66.7%) were male and 7 (33.3%) were female. Lung cancer was the most common malignancy, occurring in 11 patients (52.4%). Renal cancer was present in 2 patients (9.5%), while breast cancer, bladder cancer, myeloid sarcoma, prostate cancer, rectal adenocarcinoma, sacral chordoma, cervical cancer, and soft-tissue cancer were each present in 1 patient (4.8%).

**Figure 1:**
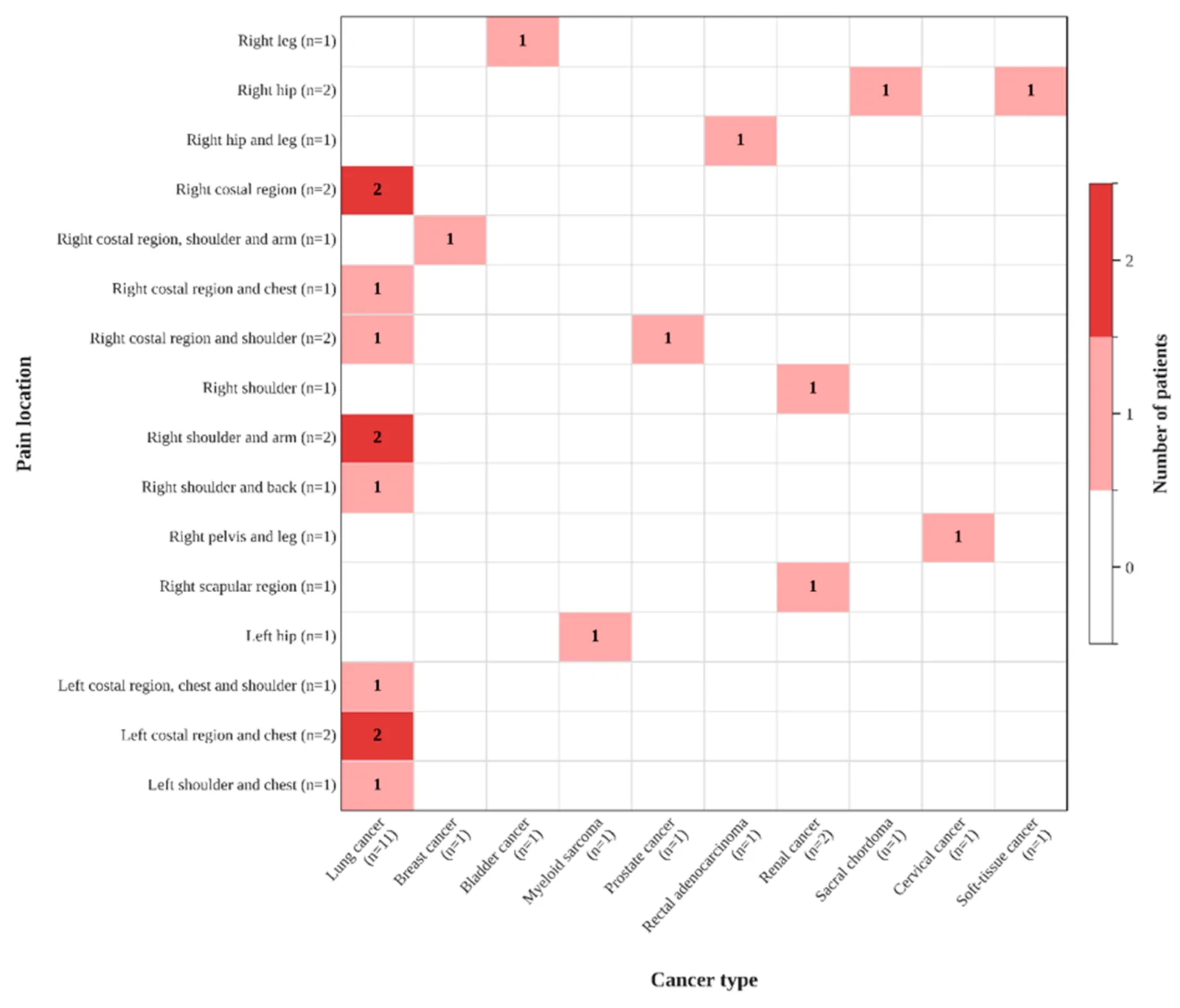
Heatmap of cancer types according to pain location. Cell values indicate the number of patients in each cancer type–pain location combination, with darker shading representing higher patient counts. Total patient numbers for each category are shown in parentheses.

Pain distribution was right-sided in 16 patients (76.2%) and left-sided in 5 patients (23.8%). The most frequently reported specific pain locations were the right hip, right ribs, right ribs and shoulder, right shoulder and arm, and left ribs and chest, each occurring in 2 patients (9.5%). Pain involving the right leg, right hip and leg, right ribs–shoulder–arm, right ribs and chest, right shoulder, right shoulder and back, right pelvis and leg, right scapula, left hip, left ribs–chest–shoulder, and left shoulder and chest was each observed in 1 patient (4.8%) (Figure 1).

**Figure 2:**
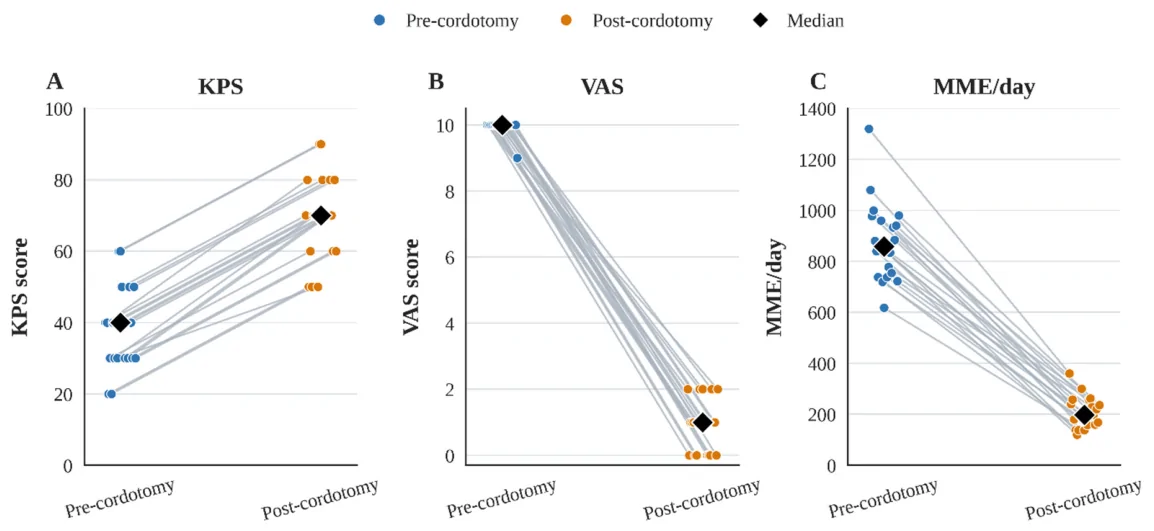
Individual paired changes in (**A**) KPS, (**B**) VAS, and (**C**) daily MME after cordotomy. Each thin gray line connects the pre- and post-cordotomy values of one patient. Blue and orange circles represent pre- and post-cordotomy measurements, respectively, while black diamonds indicate the medians. A small deterministic horizontal offset was applied to improve the visibility of overlapping trajectories without altering the measured values. KPS, Karnofsky Performance Status; VAS, Visual Analog Scale; MME, morphine milligram equivalents.

Following cordotomy, the median KPS increased from 40 to 70, while the median VAS score decreased from 10 to 1 and the mean MME/day decreased from 876.00 to 205.62. All three changes were statistically significant (all *p* < 0.001) and were associated with large effect sizes. The mean relative reduction in MME/day was 76.58 ± 5.16%. Furthermore, all three outcome measures improved in all 21 patients (100%; exact 95% CI: 83.9–100%). The paired changes in KPS, VAS, and MME/day, together with descriptive statistics, 95% confidence intervals, and corresponding *p*-values, are presented in Table 1, while individual patient-level changes are illustrated in Figure 2.

Procedure-related adverse events documented during the 1-month follow-up included transient head and neck pain in 10 patients, urinary retention in 3, transient gait disturbance in 2, and contralateral/mirror pain in 3. No permanent or major complications were documented during the standardized 1-month follow-up.

## 4. Surgical Technique and Anatomical Considerations

**Figure 3:**
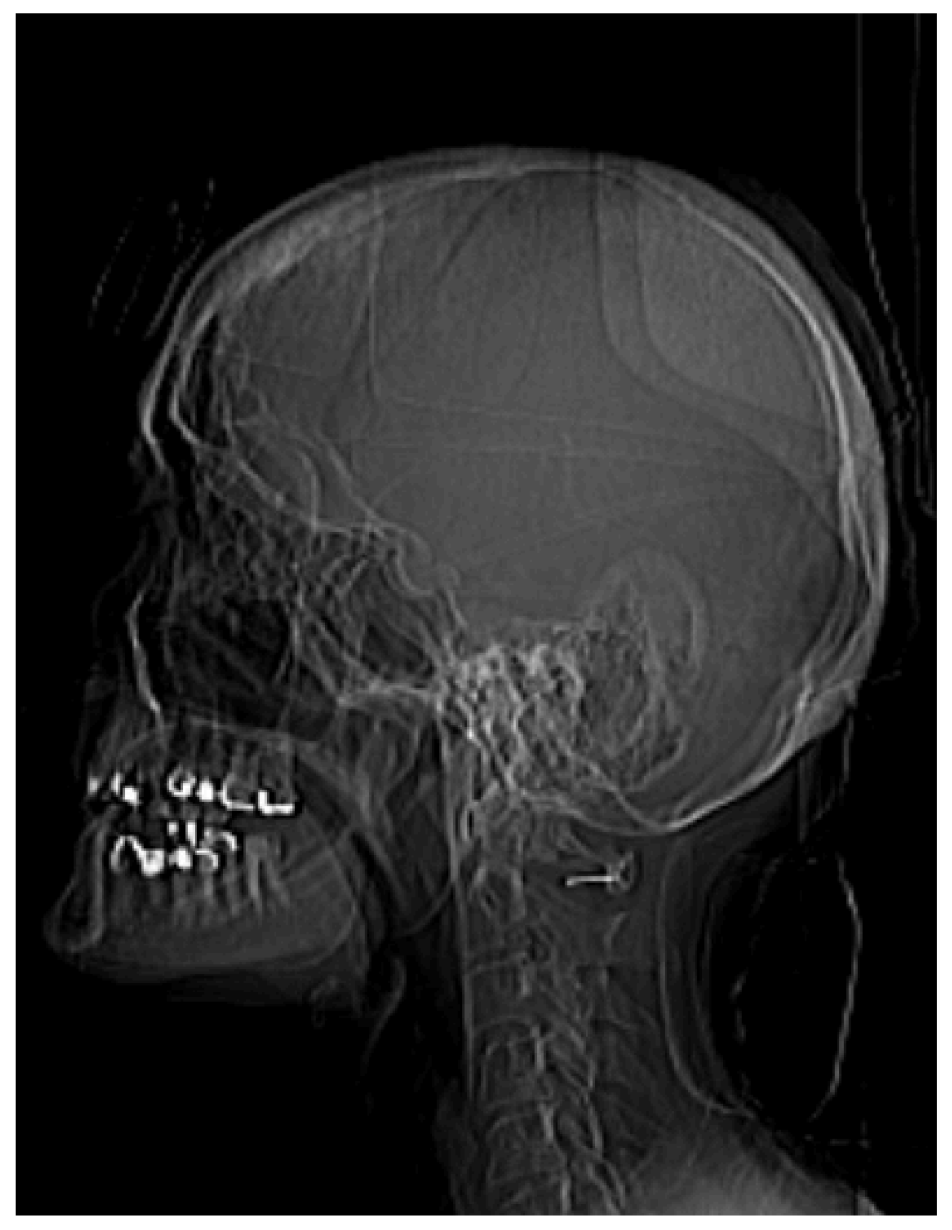
Lateral CT image demonstrating the needle position at the C1–C2 level.

Patients were positioned supine with cervical flexion maintained at 10–20° and the head securely immobilized. The mastoid process contralateral to the side of pain was identified by palpation as the primary anatomical reference point. Local anesthesia was administered subcutaneously just inferior to the mastoid process, corresponding to the C1–C2 vertebral level. A spinal needle was inserted perpendicular to the skin surface, with initial positioning at the C1–C2 level confirmed by lateral CT imaging (Figure 3).

**Figure 4:**
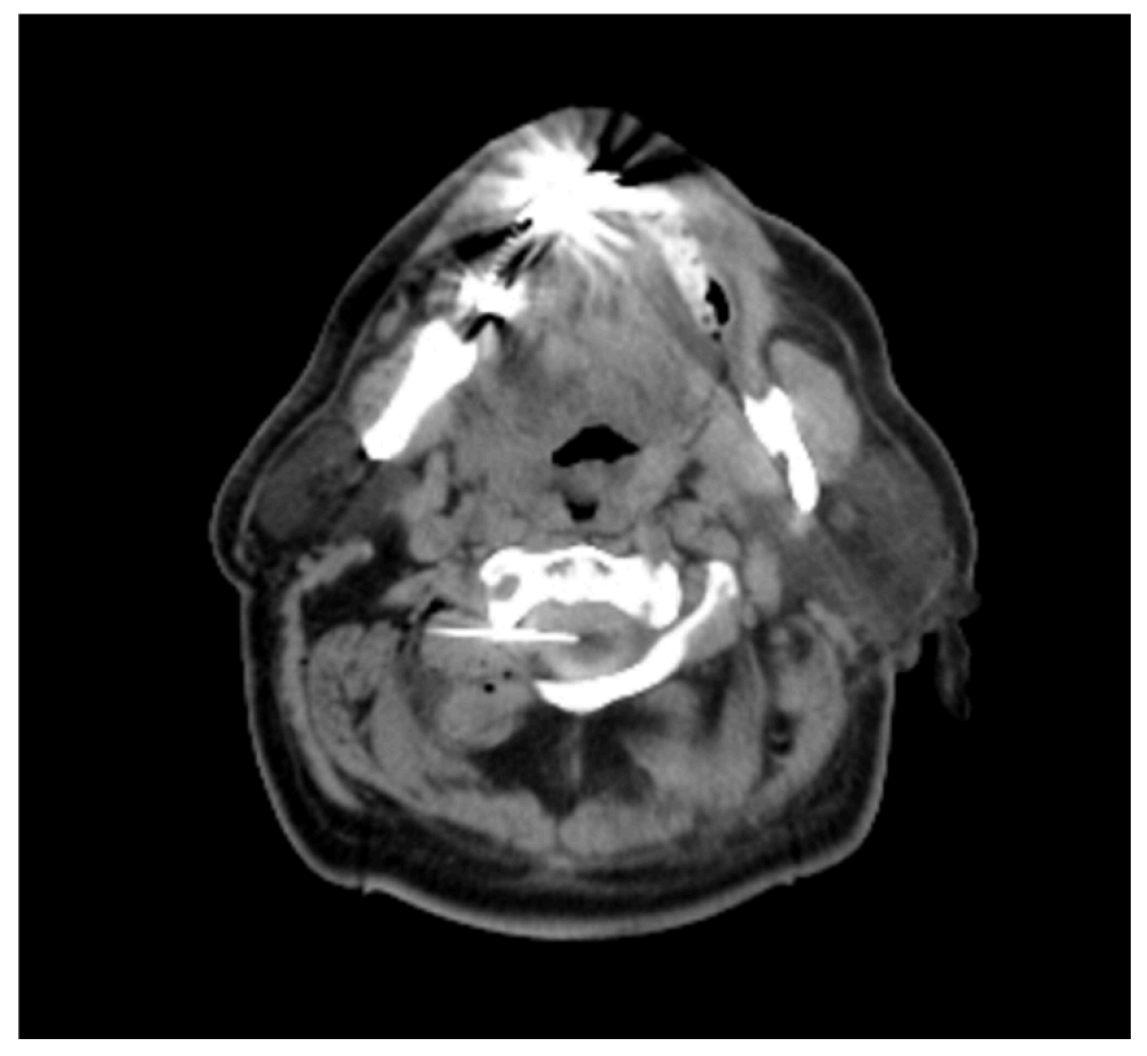
Post-iohexol image showing the needle’s final position within the anterolateral aspect of the spinal cord.

The needle trajectory, position, and proximity to the spinal cord were assessed using serial CT imaging. The target structure, the lateral spinothalamic tract (LSTT), was localized within the anterolateral quadrant of the spinal cord. As the needle approached the dura mater, the stylet was removed, and successful dural puncture was confirmed by spontaneous cerebrospinal fluid egress. Spinal cord visualization was then enhanced by intrathecal administration of a contrast solution consisting of 7 mL iohexol (Omnipaque 350 mg/mL) combined with 3 mL normal saline, injected slowly under CT guidance. Axial CT imaging demonstrated the spinal cord surrounded by contrast-enhanced subarachnoid space (Figure 4).

Following contrast administration, the radiofrequency electrode was carefully advanced through the needle under continuous impedance monitoring. Electrode advancement was monitored using serial CT imaging, and final positioning within the anterolateral aspect of the spinal cord at the LSTT target was verified by CT visualization and impedance measurements.

Electrode positioning was confirmed through systematic electrophysiological testing. Sensory stimulation at 100 Hz was performed to verify LSTT engagement, with appropriate positioning indicated by patient reports of contralateral paresthesia or thermal sensation in the painful region at low voltage thresholds (0.1–0.2 V). Higher voltage requirements prompted minor electrode repositioning. Motor stimulation at 2–5 Hz was performed to exclude proximity to motor pathways. Ipsilateral muscle contractions in the upper or lower extremities indicated excessive proximity to the corticospinal tract and prompted anterior electrode repositioning. Ipsilateral trapezius or cervical muscle contractions suggested proximity to anterior horn cells and prompted slight electrode withdrawal. The absence of motor responses between 0.5 and 2.0 V supported appropriate positioning.

Once optimal electrode positioning was achieved, thermal lesioning was performed under light sedation with propofol 0.5 mg/kg. The electrode tip was heated to 80 °C using a standardized incremental protocol: an initial 10-s lesion, followed by a 20-s extension and a final 30-s interval. After each thermal application, sensory function was assessed by pinprick testing, and ipsilateral motor strength was examined to confirm preservation of motor function.

### Statistical Analysis

Statistical analyses were performed using IBM SPSS Statistics version 32.0 (IBM Corp., Armonk, NY, USA). Numerical variables were presented as mean ± standard deviation, median (Q1–Q3), and minimum–maximum, while categorical variables were presented as numbers and percentages. Normality was assessed using the Shapiro–Wilk test. In the overall sample, a paired-samples *t*-test was used for MME/day because the paired differences were normally distributed. For the ordinal KPS and VAS variables, the two-sided exact Wilcoxon signed-rank test was applied. For MME/day, the mean difference, 95% confidence interval (CI), and Cohen’s dz effect size were reported; for KPS and VAS, the median change, bootstrap 95% CI based on 10,000 resamples, and rank-biserial correlation coefficient were reported. Figures were generated using Python 3.13.5. in the Google Colaboratory environment; small horizontal offsets were applied solely to improve the visibility of overlapping paired observations without altering the measured values. All tests were two-sided, and *p* < 0.05 was considered statistically significant.

## 5. Discussion

Percutaneous cordotomy performed under CT guidance is an interventional option for severe, unilateral cancer-related pain that is refractory to conventional medical therapy and less invasive interventional approaches [8,9]. The procedure aims to interrupt nociceptive transmission by creating a targeted thermal lesion within the lateral spinothalamic tract (LSTT), which carries pain and temperature sensation. Because spinothalamic fibers decussate after entering the spinal cord, cordotomy produces contralateral analgesia and is generally used for unilateral pain below the C5 dermatome; it is not appropriate for midline, facial, cervical, or diffuse bilateral pain syndromes. In our cohort, lung cancer was the most common primary malignancy (52.4%), consistent with previous reports in which thoracic malignancies have represented an important indication for cordotomy [5,10,11].

Previous studies have reported substantial pain reduction after cordotomy in patients with refractory cancer pain. Kanpolat et al. described favorable pain outcomes in a large series that included patients with lung cancer [2], while Ischia et al. reported pain relief in patients with Pancoast tumors and other thoracic malignancies [12]. Other series and reviews have likewise described clinically meaningful analgesia after cordotomy in advanced cancer populations [8,9,13,14,15,16]. In the present study, all patients demonstrated marked reductions in pain intensity at the standardized 1-month follow-up; however, the small and heterogeneous sample does not permit cancer-specific conclusions.

The improvement in KPS scores observed after cordotomy may reflect several factors, including pain relief and reduced opioid requirements. High-dose opioid therapy may contribute to adverse effects such as constipation, nausea, appetite suppression, and somnolence, which can impair function. Reductions in opioid dosage after cordotomy have also been reported in previous studies [15,16]. However, because this retrospective case series had no control group and assessed standardized outcomes only at 1 month, the observed functional improvement cannot be attributed solely to cordotomy, and longer-term effects were not evaluated.

The potential for significant complications remains an important consideration in cordotomy and underscores the need for careful patient selection and meticulous procedural technique. In our case series, three of 21 patients (14.3%) developed mirror pain after the procedure. This pain occurred on the side opposite the original cancer pain and was therefore ipsilateral to the cordotomy lesion. Mirror pain was tolerable in all three patients and resolved within the first month without additional intervention. Although historically attributed to unmasking of pre-existing pain, other mechanisms, including contralateral spinal cord plasticity, have also been proposed [17,18].

Additional complications observed in our series included headache or neck pain, urinary retention, and mild gait disturbance. Headache and neck pain were the most frequent complications, occurring in 10 patients; these symptoms were managed with nonsteroidal anti-inflammatory drugs and paracetamol and resolved within the first postoperative week. Three patients developed urinary retention requiring temporary urinary catheterization; the catheters were removed within 13 days after resolution. Gait disturbance occurred in two patients and resolved spontaneously within approximately 3 days. No permanent or major complications were documented during the available 1-month follow-up.

In the early era of cordotomy, severe complications were more frequently reported than in contemporary series. Historical reports described sequelae of spinal cord lesions including permanent urinary retention, incontinence, paresis, motor weakness, and loss of ambulation [19]. Sleep apnea has also been reported after cordotomy [19,20,21,22,23], underscoring the importance of precise lesion placement and careful procedural monitoring.

The adoption of CT guidance for percutaneous cordotomy offers technical advantages over historical fluoroscopy-guided approaches. CT imaging provides detailed visualization of spinal anatomy and enables monitoring of needle position, trajectory, and depth throughout the procedure. CT guidance also permits the use of water-soluble contrast agents. In historical fluoroscopy-guided cordotomy, the oil-based contrast agent Lipiodol was used to facilitate visualization of the dentate ligament. Although the dentate ligament is not visualized with water-soluble contrast, this landmark is not required for CT-guided targeting of the anterolateral spinal cord [10].

Water-soluble contrast avoids the prolonged retention associated with historical oil-based myelographic agents. Case reports and experimental literature have described arachnoiditis after myelography with oil-based contrast agents [24,25,26,27]. Lipiodol is slowly absorbed and may become encapsulated, potentially contributing to inflammatory changes and, in rare cases, pseudotumor formation. Although Lipiodol retains accepted applications outside the neuraxial space, its historical neuraxial use has been associated with inflammatory complications [27].

Severe delayed complications related to historical oil-based contrast have also been reported, including paraplegia associated with Lipiodol-induced arachnoiditis after fluoroscopy-guided cordotomy [27]. These reports support the use of contemporary imaging and contrast techniques while emphasizing that cordotomy remains an invasive procedure with potentially serious complications.

The present study has several limitations. First, the small sample size and retrospective design limit the generalizability of the findings. Second, the absence of a control group precludes causal attribution and direct comparison with alternative treatment strategies. Third, heterogeneity in primary tumor sites and metastatic locations may have influenced outcomes. Finally, standardized follow-up was limited to 1 month; therefore, the study cannot assess the durability of analgesia, pain recurrence, delayed neuropathic pain, or late complications.

## 6. Conclusions

In this retrospective single-center case series of carefully selected patients with severe, treatment-refractory cancer pain, CT-guided percutaneous cordotomy was associated with marked short-term reductions in pain intensity and opioid requirements and an improvement in functional status at one month. However, given the small sample size, absence of a control group, and potential influence of concurrent palliative treatments and other confounding factors, these findings should be considered preliminary and should not be interpreted as establishing a causal effect of cordotomy. Larger prospective, controlled, multicenter studies with longer follow-up are needed to confirm these findings, determine comparative effectiveness and durability of benefit, and more accurately characterize uncommon but potentially serious complications.

## Figures and Tables

**Table 1:** Distribution and statistical analysis of paired changes following cordotomy.

Variables	Mean Change	SD	Median Change	IQR (Q1–Q3)	Range (Min–Max)	95% CI	*p*
**KPS**	+31.43	4.78	+30	30–30	20–40	30–30	<0.001
**VAS**	−9.05	0.86	−9	−10–−9	−10–−7	−10–−9	<0.001
**MME/day**	−670.38	121.15	−660	−736–−572	−960–−480	−725.53–−615.24	<0.001

Abbreviations: KPS, Karnofsky Performance Status; VAS, Visual Analog Scale; MME, morphine milligram equivalents; SD, standard deviation; IQR, interquartile range; CI, confidence interval.

## Data Availability

The data that support the findings of this study are available from the corresponding author upon reasonable request.
